# Extracts and Gleanings

**Published:** 1872-09

**Authors:** 


### PART II.

### Abridged Extracts and Gleanings from our Exchanges

### *MULTUM IN PARVO.*

Treatment of Cancrum Oris.——Dr. McGreevy (British Medical Journal) says: "Of all the local remedies or applications I have resorted to in such cases, I have never found any application so useful or so effective as hydrochloric acid. Neither nitric acid, nitrate of silver, nor chlorate of potash, nor any other remedy that I have ever tried or used, except hydrochloric acid, did I ever find to be of the least use to check cancrum oris. I have almost never found hydrochloric acid fail to check the progress of this dreadful disease at once, and bring on a most rapid and healthy action in the part. Nor does it cause so much pain and suffering to the little patient as one would suppose, seeing that the gangrenous spot is almost entirely without feeling at the time. This acid is easily applied to the ulcer by means of a feather or small camel-hair brush. I have cured many cases of cancrum oris by this means."—*Medical Archives.—Chicago Medical Journal.*

Nitrate of Silver in Croup.—M.M. Mouttet and Rosiere, of Montepellier, report that they have cured croup, when tracheotomy was refused by the patients as a useless cruelty, by repeated insufflations of powdered nitrate of silver. The application was at once followed by the detachment and expulsion of false membrane, and was repeated a quarter of an hour after with a similar result. Three or four hours later, another insufflation was followed by so much relief that hope was entertained, and the child from this time made a steady recovery.—*Medical World.*

Perfumed Cod-Liver Oil.—Take of the essence of eucalyptus fifteen minims, cod-liver oil three ounces, and put into a well corked bottle. The fish-oil thus loses its nauseous taste. For brown oils the quantity of the essence may be increased.

The Range Carbolic Acid.—Surgeon J. H. Bill, U. S. A., opens out in the July number of the American Journal of Medical Sciences with a paper on "Carbolic Acid, its Composition, Properties, Uses in Surgery and as an Internal Remedy. The paper closes with the following summary:

From the facts laid before the reader, he thinks that these conclusions are justified—

1. It is not proven that carbol is a general disinfectant.
2. It is of the greatest use to disinfect wounds.
3. It accomplishes this (*a*) by destroying pus, etc.; (*b*) by preventing inflammation.
4. Its use in wounds moderates pain.
5. Its use on the skin relieves itching, and produces an anæsthesia sufficient for minor cutting operations.
6. It seems to be of use internally, in certain cases, in scaly skin diseases, and at least as a moderator of pain in cancer.
7. It has not proved of decided use in other diseases.

A Mild Laxative.—Dr. David Page, in a communication to the May number of the Practitioner, says: The want of a mild, but effective aperient, of convenient form, and without any of the disagreeable concomitants of most preparations of this class, frequently confronts the physician when he casts about him to meet a case of simple constipation with what he cannot readily discover, a pleasant remedy.

He recommends the following preparation of the Prussian pharmacopeia:

| | |
|---|---|
| R.—Senna leaves, . . . | oz vj. |
| Liquorice root, . . . | oz vj. |
| Fennel seeds, . . . . | oz iij. |
| Sulphur, . . . . | oz iij. |
| Refined sugar, . . . | oz xviij. |

The usual dose is a small teaspoonful at bed-time, in water, with which it is easily mixable, forming an agreeable draught. Children readily take it, with the belief that it is a sweetmeat.

Etiology of Phthisis.—In a very elaborate article, based upon careful experiments, which appeared in the last number of Virchow's Archiv, by Dr. Sommerbrodt, of Breslau, on the question "has blood, which has escaped into the air-passages, any etiological effect in the production of pulmonary tuberculosis?" the writer decides that it is only one among the many predisposing causes in tuberculous individuals, and that it acts by setting up catarrhal pneumonia.

